# A New Model for Providing Cell-Free DNA and Risk Assessment for Chromosome Abnormalities in a Public Hospital Setting

**DOI:** 10.1155/2014/962720

**Published:** 2014-07-02

**Authors:** Robert Wallerstein, Andrea Jelks, Matthew J. Garabedian

**Affiliations:** ^1^Department of Pediatrics, Santa Clara Valley Medical Center, San Jose, CA 95128, USA; ^2^Maternal Fetal Medicine, Department of Obstetrics and Gynecology, Santa Clara Valley Medical Center, San Jose, CA 95128, USA

## Abstract

*Objective*. Cell-free DNA (cfDNA) offers highly accurate noninvasive screening for Down syndrome. Incorporating it into routine care is complicated. We present our experience implementing a novel program for cfDNA screening, emphasizing patient education, genetic counseling, and resource management. *Study Design*. Beginning in January 2013, we initiated a new patient care model in which high-risk patients for aneuploidy received genetic counseling at 12 weeks of gestation. Patients were presented with four pathways for aneuploidy risk assessment and diagnosis: (1) cfDNA; (2) integrated screening; (3) direct-to-invasive testing (chorionic villus sampling or amniocentesis); or (4) no first trimester diagnostic testing/screening. Patients underwent follow-up genetic counseling and detailed ultrasound at 18–20 weeks to review first trimester testing and finalize decision for amniocentesis. *Results*. Counseling and second trimester detailed ultrasound were provided to 163 women. Most selected cfDNA screening (69%) over integrated screening (0.6%), direct-to-invasive testing (14.1%), or no screening (16.6%). Amniocentesis rates decreased following implementation of cfDNA screening (19.0% versus 13.0%, *P* < 0.05). *Conclusion*. When counseled about screening options, women often chose cfDNA over integrated screening. This program is a model for patient-directed, efficient delivery of a newly available high-level technology in a public health setting. Genetic counseling is an integral part of patient education and determination of plan of care.

## 1. Introduction

Cell-free DNA (cfDNA) is a newly available technology that allows highly accurate screening for the most common chromosome abnormalities without invasive testing. This testing identifies fetal DNA in the maternal circulation and is considered to have a detection rate for trisomy 21 and trisomy 18 of greater than 97% and greater than 80% for trisomy 13 [1–8]. Currently, consideration of cfDNA testing is recommended for women at increased risk for chromosome abnormalities including women of advanced maternal age (AMA), with abnormal serum screening results, ultrasonographic findings suggestive of aneuploidy, or history of a prior pregnancy affected by trisomy [9]. However, the utilization of this new technology and the specifics of incorporating it into routine care are complex, as the information obtained from cfDNA screening may overlap or contradict that from maternal serum screening, nuchal translucency ultrasound, or the genetic sonogram.

We present our experience with implementing a new program for cfDNA screening in a public hospital setting with attention to patient education and early genetic counseling to individualize care and eliminate redundant screening. The aim of this report is to assess implementation of this program in terms of diagnostic testing elected by participating patients in comparison to a cohort of AMA patients seen prior to availability of cfDNA in our practice. In addition, we sought to analyze concurrent trends in prenatal ultrasound practice, specifically whether nuchal translucency utilization and/or the relative importance of ultrasound soft markers at the detailed ultrasound changed after integration of cfDNA.

## 2. Methods

In response to the availability of cfDNA, beginning in January 2013, we implemented a new patient care program entitled advanced maternal age options (AMA Options) to incorporate cfDNA testing into the existing prenatal diagnosis services at Santa Clara Valley Medical Center (SCVMC) Health and Hospital System. SCVMC is a tertiary care public health hospital with 6 free standing ambulatory health centers providing a full scope of maternal child health services. In addition, there are multiple community partner clinics that refer high-risk women for specialized pregnancy, delivery, and neonatal care. Genetics and maternal fetal medicine ultrasound and consultation services are provided together at a single centralized ambulatory clinic location, which is also designated as a regional prenatal diagnosis center certified by the State of California Department of Health Genetic Disease Screening Program. Genetic counseling is provided by licensed genetic counselors in the patient's preferred language, either with the aid of native speaking counselors or professional translators. Our system provides care to a predominantly Hispanic population (74.0% in 2012; California Maternal Quality Care Collaborative Maternal Data Center; accessed 5 November 2013), as well as a significant number of women of Asian/Pacific Islander decent (13.4% in 2012).

The goal of the AMA Options program was to create a patient-directed plan of care for high-risk women that would allow the greatest access to a variety of testing options and avoid performing redundant screening. Women who were identified as high risk (aged 35 or older at delivery or those with a prior family history of trisomy 13, 18, or 21) were referred for genetic counseling by their primary obstetricians during the late first trimester, ideally between 11 and 12 weeks of gestation. Genetic counselors reviewed the available testing options including cfDNA, first and second trimester serum screening, nuchal translucency (NT) ultrasound, detailed ultrasound, and amniocentesis. During that appointment, a patient-directed plan of care was created according to one of four care pathways: (1) cfDNA; (2) integrated screening (first trimester serum screening with NT ultrasound and second trimester quad screening); (3) direct-to-invasive testing (chorionic villus sampling (CVS) or amniocentesis); or (4) no screening. cfDNA or first trimester serum screening was performed during that visit, if desired. We assumed that this schema of stratifying choices would allow women to choose which testing they preferred while avoiding performance of multiple testing modalities on the same woman (i.e., women would not get first trimester serum screening and NT ultrasound if they were electing cfDNA).

Women were by and large covered through the California medical program which recognized cfDNA as a covered service for high-risk pregnancies. The Harmony Prenatal Test (Ariosa Diagnostics, San Jose, California) was available and, in most cases, was a covered benefit. Results of the cfDNA took approximately 10 days. Women did not have direct access to cfDNA screening without counseling through this program.

All participating women were scheduled for detailed ultrasound between 16 and 22 weeks (ideally between 18 and 20 weeks). Women were seen briefly for a second genetic counseling appointment in coordination with their detailed ultrasound to review the results of first trimester risk assessment and finalize their decision for amniocentesis. If desired, amniocentesis was performed in conjunction with the detailed ultrasound. Second trimester AFP screening for neural tube defects was offered to all patients not having amniocentesis performed. All first and second trimester serum specimens were processed through the California Genetics Disease Screening Program.

We compared high-risk women seen in the AMA Options program between January and September 2013 to advanced maternal age women seen in our clinic during the same period in 2012, prior to the initiation of the AMA Options program and prior to availability of cfDNA in our practice. During 2012, high-risk women were generally offered standard first and second trimester screening by their primary obstetricians and referred and seen for genetic counseling in the second trimester (ideally at 18 weeks) on the same day and immediately prior to a detailed ultrasound, with amniocentesis, if desired. NT ultrasound was offered and scheduled between 11 and 14 weeks as available. Women who had abnormal screening were seen for genetic counseling within 5 days. They were offered CVS (prior to 14 1/7 weeks) or amniocentesis (after 16 0/7 weeks) and detailed ultrasound. For nuchal translucency ≥3.5 mm, patients were also offered fetal echocardiography between 20 and 22 weeks. As all women with screen positive results were covered through California public insurance, their out-of-pocket expense was not a determining factor in test choices.

Choices in prenatal diagnostic testing and indications for invasive testing among all patients seen for genetic counseling prior to and following initiation of the AMA Options program were recorded by the genetic counselors in a prospective interdepartmental database. Indication for invasive testing was classified as fetal anomaly (if any fetal anomalies other than soft markers were found at the time of the detailed ultrasound); ultrasound soft marker (see below); abnormal serum screening (screen positive on first and/or second trimester screening); family history (patient or 1st degree relative with congenital anomaly, mental retardation, genetic syndrome, or aneuploidy); or advanced maternal age only (if absence of any of the above indications).

The presence of fetal anomalies or ultrasound soft markers was recorded by the perinatologist performing the detailed ultrasound. Ultrasound protocols in use by our department during both 2012 and 2013 specified reporting of 6 soft markers in patients undergoing either standard or detailed sonograms between 16 and 22 weeks: echogenic intracardiac focus (unilateral or bilateral, isoechoic to bone) [10]; choroid plexus cyst (unilateral or bilateral, >5 mm diameter) [10]; echogenic bowel (isoechoic to bone) [11]; pyelectasis (renal pelvis ≥4 mm) [12]; shortened humerus (less than 2.5% percentile for BPD) [13]; and nuchal thickness ≥6 mm (on angled axial view of upper cerebellum) [11]. Management and patient counseling upon finding of one or more soft markers was individualized by the perinatologist performing the ultrasound.

The Santa Clara Valley Medical Center Institutional Review Board granted exemption from review for this project. Statistical analysis was performed using STATA/IC 12 (StataCorp, College Station, TX) to test for differences in proportions.

## 3. Results

In the first 9 months of 2013, 181 women were seen in our unit for AMA Options counseling. For 163 (90%) of these, complete information about ultrasound findings and diagnostic testing choices at the 16–22-week detailed ultrasound were available, and these women are the subject of the current report. Of those who did not follow up for second trimester detailed ultrasound in our unit, there was no difference in patient demographics (age 38.0 versus 38.6 years; gestational age 11.5 versus 12.1 weeks), proportion choosing early cfDNA screening (66.7%) or invasive testing (16.7%), or proportion with abnormal screening (0.0%).

**Figure 1:**
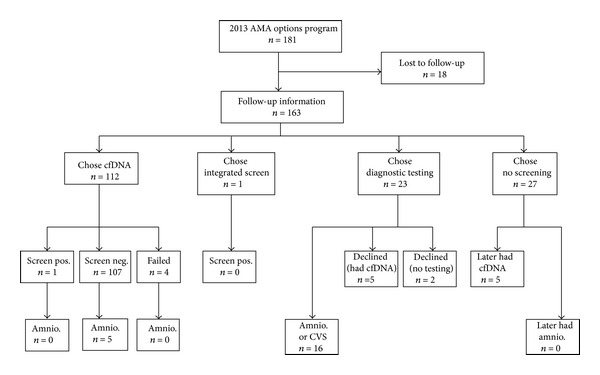
Patient eligibility for AMA Options genetic counseling and choice of testing strategy.

Figure 1 shows the number of women electing the various options presented at the AMA Options counseling session: cell-free fetal DNA screening (112/163, 68.7%), integrated screening (1/163, 0.6%), direct-to-invasive testing (23/163, 14.1%), and no screening (27/163, 16.6%). Of those who initially chose no screening, 5/27 (18.5%) women ultimately did desire and underwent cfDNA; and, of those who initially elected direct-to-invasive testing, 5/23 (21.7%) ultimately chose to undergo cfDNA screening in lieu of amniocentesis in the second trimester. Overall, a total of 122 women (122/163, 74.8%) underwent cfDNA.

One woman (1/122, 0.8%) was screened positive for trisomy 21 on cfDNA but did not elect invasive testing. She experienced a spontaneous abortion of dichorionic twins at 17 weeks; genetic testing was not performed on the conceptus. Four women (4/122, 4.1%) failed to obtain a result from cfDNA, none of whom underwent invasive testing. Three of these women had normal detailed ultrasounds. The fourth experienced a fetal demise at 15 weeks; karyotype and microarray were normal on the products of conception.

Table 1 shows the 16–22-week ultrasound findings and diagnostic testing elected for women who had undergone AMA Options counseling and the reasons cited for invasive testing. A total of 21/161 (13.0%) women ultimately underwent invasive testing; 16/23 had initially selected this as their preferred testing method. Five additional women elected amniocentesis after normal cfDNA testing and normal detailed ultrasound. Two women chose CVS, one of whom had an unsuccessful procedure and ultimately underwent amniocentesis; a total of 20 amniocenteses were performed. No abnormal karyotypes were found.

Two noteworthy trends in practice were observed in 2013 after initiation of the AMA Options program. We found a significant decrease in the proportion of women who had ultrasound soft markers reported during the detailed ultrasound (37/457, 8.1% versus 5/161, 3.1%, *P* = 0.03). We also noted that, when comparing our overall AMA population between 2012 and 2013, a much lower proportion of AMA women overall underwent nuchal translucency ultrasounds in 2013 after initiation of AMA Options (Table 2). Consequently, a higher proportion of available NT ultrasound appointments were allocated to patients aged less than 35.

In 2012 and 2013, a similar small proportion of patients undergoing nuchal translucency ultrasound were screened positive for trisomy 21 or 18 (Table 2), and a similar proportion was found to have nuchal measurement exceeding 3.5 mm. Only one case of congenital heart disease occurred in the group with large nuchal translucency; this case was associated with findings of multiple anomalies and confirmed 45X monosomy at 17 weeks; the patient elected pregnancy termination. Across both epochs, there was only 1 woman who had an infant with trisomy 21 without being prenatally detected. This patient was 32 years of age and had integrated serum screening with normal results.

## 4. Discussion

In the current report, we describe our experience with implementation of a novel program to incorporate cfDNA screening with patient-specific genetic counseling in a public hospital setting. We believe our AMA Options program can serve as a model for use of a newly available high-level technology in a public health setting. We believe our AMA Options program serves as a model for implementation of cfDNA in a public health setting hospital system. With implementation of this program of patient-directed aneuploidy assessment, we were able to provide first trimester genetic counseling to nearly 40% of our AMA population while minimizing redundant testing strategies. When presented with options for aneuploidy screening, nearly 70% of these patients opted for cfDNA screening and chose to forgo integrated first and second trimester screening. This is consistent with the anticipated 71.9%–79% of women expressing a desire for cfDNA testing [14, 15]. Our experience has been very different from that of Taylor et al., who offered cfDNA to all women considering genetic testing with a 28% of women opting for cfDNA over integrated screening [16]. Interestingly, 1 in 6 patients initially opted to have no screening or diagnostic testing, suggesting that a significant portion of our patients do not desire antenatal information about aneuploidy risk when provided with genetic counseling.

Integrated algorithms incorporating first and second trimester serum analytes, with and without first trimester nuchal translucency, have been developed [17]. However, these algorithms do not currently incorporate cfDNA, leaving providers and patients to face the question of whether to use cfDNA in addition to or in place of integrated screening. When combining different independent screening tests, one must be cognizant of the additive effect on false positive rates. With our AMA Options program, we have minimized the problem of a compounded false positive rate by offering patients who present for care early in pregnancy the choice of one of several discrete screening pathways. This strategy avoids simply adding a new test on top of existing options in a haphazard manner. Additionally, by providing pre- and posttest genetic counseling, in a manner consistent with ACOG guidelines [17], patients are provided with a clear understanding of rates of detection and false positive results, advantages and disadvantages of the different strategies, and the role of diagnostic procedures.

We also examined how the AMA Options program affected health care delivery within our system. A decrease in utilization of amniocentesis was observed, consistent with published experience [18]. Interestingly, we found an apparent change in practice pattern with respect to the reporting of soft markers for chromosome abnormalities during second trimester ultrasonography. Among women offered cfDNA in the first trimester, soft markers were reported less frequently. Likelihood ratios of soft markers noted on second trimester ultrasound and after first trimester, second trimester, and integrated screening have been calculated [19, 20]. The utility of these findings following cfDNA screening is currently unknown; however, given that the reported risk of selected chromosome abnormalities is 1 : 10,000 with a negative cfDNA screen, it seems unlikely that the presence of isolated soft markers on genetic ultrasound would increase the risk to a significant level. We speculate that MFM providers performing the second trimester ultrasound on women who had already had negative cfDNA testing were more reluctant to report soft markers to avoid patient confusion.

Current guidelines call for the use of cfDNA in populations considered high risk for chromosome abnormalities [9]. While cfDNA does have appealing characteristics, such as its noninvasive nature, high detection rate for the most common aneuploidies, and low false positive rate, it should be integrated into clinical practice in conjunction with appropriate counseling, to ensure that patients understand the test and its limitations [21]. Currently, ACOG recommends pretest genetic counseling to inform patients of the abilities and limitations of cfDNA [9]. Such counseling is important to guide patients through a very complex decision involving multiple tests that provide similar information. After initiation of our AMA Options program (during which most women declined first trimester screening in favor of cfDNA), we noted increased access to nuchal translucency ultrasound appointments for non-AMA (or low-risk) women. In our public health hospital system, NT appointments are a limited resource, and reallocation of these appointments has helped to further our goal to offer first trimester aneuploidy screening to all women in our system.

Presently, cfDNA screening is not recommended for use in a low-risk population, as the performance of cfDNA in these women has not been adequately evaluated. In contrast, integrated screening has been evaluated and is appropriate for use in women younger than 35 years old [22]. Existing data on the use of cfDNA in non-high-risk women is promising, but further studies are needed to better understand testing performance in low-risk or unselected populations [23]. The false positive rate of cfDNA screening is an important consideration, as acting on a positive result without confirmatory testing may lead to undesired termination of nonaneuploid fetuses [23]. As such, cfDNA must be used as a screening test and confirmatory testing is recommended to inform decisions about pregnancy termination [9, 21].

One potential criticism of our approach is that patients electing cfDNA no longer undergo formal first trimester assessment of nuchal translucency (NT). The NT ultrasound's purpose is primarily for first trimester aneuploidy risk assessment as most women have a dating ultrasound with their primary obstetric provider to assure correct scheduling. It is uncommon to detect congenital heart disease by enlarged NT alone. While a large NT has been associated with congenital heart disease [24–28], this sonographic finding is a poor screening tool for congenital heart disease. While there are multiple definitions for enlarged NT in the literature (e.g., ≥3.5 mm, >95th percentile, ≥2.0 MoM, ≥2.5 MoM, ≥3.0 MoM), all have a poor specificity (≤20%) for isolated congenital heart disease [26–28]. While the first trimester NT may be useful for identifying those fetuses at high risk for congenital heart disease, the patients enrolled in the AMA Options program are all considered sufficiently high risk for congenital anomalies that they receive a detailed second trimester ultrasound, with thorough evaluation of fetal cardiac anatomy. While there may be value in earlier detection of congenital heart disease, given its overall low prevalence in this population, we do not see the NT as a test with sufficient performance as a screening test to be an obligatory part of prenatal care for the purposes of screening for congenital heart disease.

Noninvasive prenatal testing is an evolving technology. Starting with assays of maternal serum AFP to the current era of cfDNA, integration of new technologies has presented challenges. Integrating cfDNA into current practice must be done in a rational manner and in conjunction with appropriate counseling. Patients need this counseling to help inform very difficult decision benefits, risks, and limitations of multiple alternatives for aneuploidy screening [23].

In the context of a public health hospital system, resource allocation is an important consideration. While the actual impact of cfDNA implementation on health care cost is still undetermined, recent cost-benefit analysis supports implementation in high-risk populations over other screening algorithms [23, 29–32]. Based upon a theoretical cohort of 4 million pregnancies, Song et al. demonstrate a higher detection rate and net cost savings when screening for trisomy 21 is done with cfDNA in comparison to traditional approaches. With the AMA Options program, in addition to a high acceptance and utilization of cfDNA screening, we have further been able to provide efficient care through the minimization of redundancy in prenatal diagnosis. Additionally, we have been able to improve availability of aneuploidy screening, in the form of first trimester nuchal translucency screening, to new segments of our patient population. In the era of accountable care organizations, this program furthers the goal of providing high quality care while eliminating redundancy in care provided.

The initial experience with our AMA Options program demonstrates that a rational approach to integration of cfDNA into obstetric practice is feasible and efficient. Master's level genetic counselors can provide patient education and assistance with decision making to create an individualized plan of care. Utilizing this model, our patients have embraced this new screening option. We have found that there may be unanticipated practice changes with adoption of cfDNA, specifically with a decreased frequency of reporting isolated soft markers for aneuploidy; however, the clinical impact of such change is unclear. Moving forward, other systems are encouraged to be cognizant as to how cfDNA is implemented in their systems. With AMA Options, we provide one model for how this can be done in a rational manner.

## Figures and Tables

**Table 1:** Amniocentesis utilization and reason: 2013 AMA Options verses 2012 all AMA.

	2013 AMA Options			2012 all AMA			*P* value
	*N*	Amnio./CVS, *n *	% (*n*/*N*)	*N*	Amnio./CVS, *n*	% (*n*/*N*)	
Total	**161**	21	13.0%	**457**	87	19.0%	0.08
Anomaly	0	0	0.0%	9	7	77.8%	
SM + Abnormal screen	0	0	0.0%	9	6	66.7%	
Soft marker only	5	1	20.0%	37	9	24.3%	0.83
Abnormal serum screen only	7	2	28.6%	58	18	31.0%	0.91
Family history	4	0	0.0%	16	3	18.8%	0.35
AMA only	145	18	12.4%	328	44	13.4%	0.77

**Table 2:** Nuchal translucency screen positive results and outcomes: 2012 verses 2013.

	Epoch 1: January–September 2012 *N* = 683	Epoch 2: January–September 2013 *N* = 521	*P* value
Screen positive	14 (2.0%)	13 (2.5%)	** 0.56**
Nuchal translucency ≥3.5 mm	1 (0.1%)	2 (0.4%)	**0.28**
Congenital heart disease	0 (0%)	1 (0.2%)	**0.24**
Aneuploidy (Turner's = 1)	0 (0%)	1 (0.2%)	**0.24**

Data reported as *n* (%).
